# Prevalence of potentially inappropriate medications among old people with major neurocognitive disorder in 2012 and 2017

**DOI:** 10.1186/s12877-022-03240-y

**Published:** 2022-06-30

**Authors:** Iris Rangfast, Eva Sönnerstam, Maria Gustafsson

**Affiliations:** Department of Integrative Medical Biology, Umeå University, 901 87 Umeå, Sweden

**Keywords:** Potential inappropriate medications, Adverse drug reaction, Explicit quality indicators, SveDem

## Abstract

**Background:**

The increased risk of adverse drug reactions due to age-related altered pharmacokinetics and pharmacodynamics is a challenge when prescribing medications to older people, and especially among older people with major neurocognitive disorder who are particularly sensitive to drug effects. The aim of this study was to investigate the use of potential inappropriate medications (PIMs) in 2012 and 2017 among old people with major neurocognitive disorder. A secondary aim was to investigate factors associated with PIM use.

**Methods:**

This register-study was based on the Swedish registry for cognitive/dementia disorders and the Swedish prescribed drug register. Criteria from the National Board of Health and Welfare were used to identify PIMs between 1 July–31 December 2012 and 1 July-–31 December 2017 among people ≥ 65 years. Drug use was defined as one or more filled prescriptions during each timeframe.

**Results:**

The total use of PIMs declined significantly between 2012 (28.7%) and 2017 (21.7%). All PIMs and PIM groups declined between these years, except for antipsychotic drugs, which increased from 11.6% to 12.3%. The results from the multiple regression model found that PIM use was associated with younger age (OR: 0.97 CI: 0.96–0.97), a lower Mini Mental State Examination score (OR: 0.99 CI: 0.99–1.00), the use of multi-dispensed drugs (OR: 2.05 CI: 1.93–2.18), and compared to Alzheimer’s disease, with the subtypes dementia with Lewy bodies and Parkinson’s disease dementia (OR: 1.57 CI: 1.40–1.75), frontotemporal dementia (OR: 1.29 CI: 1.08–1.54) and vascular dementia (OR: 1.10 CI: 1.03–1.16).

**Conclusions:**

Overall, the use of PIMs decreased between the years 2012 and 2017. The increase of antipsychotic drugs and the association between PIM use and multi-dispensed drugs warrant concern.

**Supplementary Information:**

The online version contains supplementary material available at 10.1186/s12877-022-03240-y.

## Introduction

One of the important challenges when treating older people with drugs is the increased risk of adverse drug reactions (ADRs) due to altered pharmacokinetics and pharmacodynamics [1]. This is further complicated among people with major neurocognitive disorder due to high prevalence of multimorbidity, drug burden and a change in neurotransmitter substances that is more pronounced compared to older people without this disorder [2–4]. The WHO estimates that 50 million people worldwide suffer from major cognitive disorder and, in Sweden, the National Board of Health and Welfare estimates the prevalence to be 130,000–150,000 people with 20,000–25,000 new incidents every year [5, 6].

Potentially inappropriate medications (PIMs) refer to the use of medication for which the associated risk outweighs the potential benefits, especially when other alternatives are available for the individual [7, 8]. The use of PIMs has been associated with an increased risk of hospitalisation and mortality in older people [9]. There are several tools available to help identify PIMs and, in Sweden, the National Board of Health and Welfare has developed specific quality indicators for older individuals [10]. The criteria were last updated in 2017, and include PIMs in general and PIMs in certain diseases. The indicators are not just to be used as a support to improve the prescription of drugs, but also as an instrument to measure and monitor the quality of drug treatment among older people [10].

Previous studies using different criteria have found that treatment with PIMs is prevalent in old people with major neurocognitive disorder [11–16]. However, to our knowledge, many studies have not investigated trends in the prevalence of PIMs in a large population including only people with major neurocognitive disorder. The Swedish registry for cognitive/dementia disorders (SveDem) is a national quality register that started in 2007 [17]. The Swedish prescribed drug register (SPDR) was started in 2005 and provides data on prescribed drugs that are dispensed at all Swedish pharmacies [18]. In this study, these two registries were combined in order to obtain data regarding PIM use in a large population with major neurocognitive disorder. The aim of this study was to investigate the use of potential inappropriate medications (PIMs) in 2012 and 2017 among old people with major neurocognitive disorder. A secondary aim was to investigate associated factors with PIM use.

## Method

### Study population

This was a nationwide register-based study based on the SveDem and the SPDR. These two registries were combined in order to investigate the use of PIMs among two cross-sectional samples of people with major neurocognitive disorders, during the periods 1 July–31 December 2012 and 1 July –31 December 2017. People who were ≥ 65 years old, registered in SveDem and with a diagnosis date no later than 30 June 2012 and alive on 31 December 2012 (*n* = 20,889), and with diagnoses dates no later than 30 June 2017 and alive on 31 December 2017 (*n* = 35,212) were included in the study. The Swedish Cause of Death Register was used to exclude people deceased before 31 December 2012 and 31 December 2017 respectively.

### The Swedish registry for cognitive/dementia disorders

SveDem is a nationwide quality registry on major neurocognitive disorders. The number of registrations is growing continuously, and 101,503 people were registered in August 2021. Information about major neurocognitive disorder diagnosis (ICD-10), basic investigation, demographic data, and medical treatment are some examples of data that are registered in the registry [17].

### The Swedish prescribed drug register

SPDR is complete for all residents in Sweden and provides data on all prescribed drugs that are dispensed at all Swedish pharmacies. All drugs are classified according to the Anatomical Therapeutic Chemical (ATC) classification system. The register includes for example data on age, sex, and information about the prescription and prescriber. Information about over-the counter drugs and drugs dispensed within other parts of health care, e.g. at hospitals are not included [18].

### Definitions

Drug use was defined as one or more filled prescriptions during each timeframe (1 July–31 December 2012 and 1 July–31 December 2017). To identify PIM substances, the quality indicators published by the Swedish National Board of Health and Welfare were used. Six drug-specific indicators were investigated; benzodiazepines with long duration, anticholinergic substances, propiomazine, codeine, glibenclamide and tramadol. According to the quality indicators, the number of people using these drugs should be as low as possible, regardless of indication. According to the same indicators, antipsychotic drugs and NSAIDs are classified as drugs for which correct and current indication is of particular importance. Since these drugs may have many side-effects in this group of people, we also included these drugs even though the indication was unknown.

### Data analysis

Pearson chi-square test was used to analyse dichotomous variables and independent sample t-test was used to analyse continuous variables. Multiple logistic regression analysis was conducted to investigate the PIM use in 2012 and 2017. The model had PIM use as the dependent variable and included age, sex, baseline Mini Mental State Examination (MMSE) score and year of investigation (2012 or 2017) as independent variables. MMSE scores between 0–30, and a score of 23 or below is generally considered to indicate cognitive impairment [19, 20].

A regression model was also performed to investigate associated factors for total PIM use. In this analysis, a subgroup based on diagnosis type was selected and people with the following four major NCD subtypes were included from both 2012 and 2017: vascular dementia (9,831 individuals), frontotemporal dementia (692 individuals), early and late Alzheimer’s disease (19,167 individuals) and subtypes associated with Lewy body pathology; dementia with Lewy bodies and Parkinson’s disease dementia (1,732 individuals). Consequently 24,679 people with mixed, unspecified and other dementias were excluded. The model had PIM use as the dependent variable and age, sex, year of investigation, multi-dispensed drugs, baseline MMSE score and the four major neurocognitive disorders as independent variables. Significant variables from the simple logistic regression analysis, as well as sex and age, were included in a multiple logistic regression analysis. Results were presented as odds ratio (OR) with 95% confidence interval (CI) and a p-value < 0.05 was considered statistically significant. IBM SPSS Statistics version 26 and SAS Enterprise Guide 7.1 were used for all handling, calculations, and analyses of the data.

## Results

The basic characteristics of the population are summarised in Table 1. The population included 20,899 individuals in 2012 and 35,212 individuals in 2017. There was a significant difference in age between the years, 81.9 (± 6.5) years in 2012 and 82.7 (± 6.6) years in 2017. There was also a difference regarding the number of people using multi-dispensed drugs in 2012 (50.7%) and in 2017 (64.1%). Further, among the types of major neurocognitive disorders, Alzheimer’s disease with early and late onset was most common in both 2012 (33.1%) and 2017 (34.8%), and the difference between the years was significant.

**Table 1:** An overview of the basic characteristics in the population in 2012 and 2017

Basic characteristics	2012	2017	*p*-value
***Study population, n***	**20,889**	**35,212**	
Age (mean ± SD)	81.9 ± 6.5	82.7 ± 6.6	< 0.001
Females^a^, n (%)	12,801 (61.3%)	21,746 (61.8%)	0.254
Multi-dispensed drugs, n (%)	10,590 (50.7%)	22,564 (64.1%)	< 0.001
Baseline MMSE-score^b^ (mean ± SD)	21.5 ± 4.9	21.5 ± 4.7	0.755
*Type of major neurocognitive disorder, n (%)*			
Early and late onset Alzheimer’s disease	6,915 (33.1%)	12,252 (34.8%)	< 0.001
Vascular dementia	3,506 (16.8%)	6,325 (18.0%)	< 0.001
Mixed Alzheimer’s disease and vascular dementia	3,728 (17.8%)	6,470 (18.4%)	0.117
Dementia with Lewy bodies	392 (1.9%)	605 (1.7%)	0.170
Parkinson’s disease dementia	291 (1.4%)	444 (1.3%)	0.183
Frontotemporal dementia	247 (1.2%)	445 (1.3%)	0.399
Unspecified	5,356 (25.6%)	7,796 (22.1%)	< 0.001
Other dementia type	454 (2.2%)	875 (2.5%)	0.019

*MMSE* Mini Mental State Examination, *SD* standard deviation

^a^Missing information in 3 (2012) and 10 (2017) cases

^b^Missing information in 1,663 (2012) and 1,590 (2017) cases

The results from the analysis of PIM use between 2012 and 2017 are presented in Table 2. Overall, the use of PIMs had decreased 2017 (21.7%) compared to 2012 (28.7%) (*p* < 0.001) and almost all subgroups of PIM decreased significantly between these years. For example, anticholinergic drugs decreased from 9.1% to 6.0% (*p* < 0.001), and NSAID from 4.9% to 2.7% (*p* < 0.001). On the contrary, antipsychotic drugs increased between 2012 (11.6%) and 2017 (12.3%) (*p* < 0.001). Antipsychotic drugs were also the most commonly used drug group in both 2012 and 2017. The prevalence in 2012 and 2017 for each specific substance are provided in Additional file 1: Table S1.

**Table 2:** Multiple logistic regression analysis of PIM use in each drug/drug group between 2012 and 2017

Drug groups	2012	2017	OR^a^ (95% CI)	*p*-value
*All people, n*	20,889	35,212		
Benzodiazepines, n (%)	434 (2.1)	511 (1.5)	0.731 (0.637–0.837)	< 0.001
Anticholinergics, n (%)	1898 (9.1)	2098 (6.0)	0.657 (0.614–0.702)	< 0.001
Tramadol, n (%)	367 (1.8)	114 (0.3)	0.195 (0.157–0.242)	< 0.001
Propiomazine, n (%)	608 (2.9)	352 (1.0)	0.365 (0.318–0.419)	< 0.001
Codeine, n (%)	546 (2.6)	408 (1.2)	0.440 (0.385–0.502)	< 0.001
Glibenclamide, n (%)	197 (0.9)	87 (0.2)	0.269 (0.208–0.349)	< 0.001
NSAIDs, n (%)	1024 (4.9)	941 (2.7)	0.562 (0.512–0.617)	< 0.001
Antipsychotics, n (%)	2431 (11.6)	4323 (12.3)	1.148 (1.085–1.215)	< 0.001
PIM (total), n (%)	5995 (28.7)	7629 (21.7)	0.724 (0.695–0.754)	< 0.001

*CI* confidence interval, *NSAIDs* non-steroidal anti-inflammatory drugs, *OR* odds ratio, *PIM* potentially inappropriate drug

^a^Adjusted for sex (missing in 3 (2012) and 10 (2017) cases), age and baseline Mini Mental State Examination score (missing in 1,663 (2012) and 1,590 (2017) cases), reference category year 2012

Factors associated with the total PIM use are presented in Table 3. Filled prescriptions for total PIMs were negatively associated with the year 2017. The use of multi-dispensed drugs, younger age and lower MMSE were also associated with PIM use. Furthermore, the result indicated an association of PIM use in the diagnoses vascular dementia, dementia with Lewy bodies/Parkinson’s disease dementia, and frontotemporal dementia compared with the reference category Alzheimer’s disease. Regarding the association between sex and PIM, the result was not statistically significant in the simple or in the multiple regression analysis.

**Table 3:** Simple and multiple logistic regression analysis of factors associated with PIM use

**PIM**	**Simple OR**	**95% CI**	***P*-value**	**Multiple OR**	**95% CI**	***P*-value**
Female sex^a^	1.020	0.967–1.076	0.459	0.991	0.935–1.050	0.758
Older age	0.979	0.975–0.983	< 0.001	0.968	0.964–0.972	< 0.001
Multi-dispensed drugs	1.768	1.675–1.867	< 0.001	2.052	1.932–2.179	< 0.001
Baseline MMSE-score^b^	0.987	0.982–0.993	< 0.001	0.992	0.987–0.998	0.007
Year 2017 ^c^	0.687	0.652–0.724	< 0.001	0.652	0.617–0.690	< 0.001
Vascular dementia^d^	1.145	1.082–1.212	< 0.001	1.095	1.030–1.164	0.003
Dementia with Lewy bodies and Parkinson dementia^d^	1.755	1.580–1.950	< 0.001	1.567	1.401–1.753	< 0.001
Frontotemporal dementia^d^	1.465	1.241–1.730	< 0.001	1.292	1.081–1.544	0.005

*CI* confidence interval, *MMSE* Mini Mental State Examination score, *OR* odds ratio, *PIM* potentially inappropriate medication. PIM use was the dependent variable. Sex, age, multi-dispensed drugs, MMSE, year of investigation and type of major neurocognitive disorder were independent variables

^a^Data were missing for 6 individuals

^b^Data were missing for 1,457 individuals

^c^Reference category year 2012

^d^Reference category Alzheimer’s disease (early and late onset)

## Discussion

The main finding of this study was that, overall, the use of PIMs declined significantly between 2012 (28.7%) and 2017 (21.7%) among people with major neurocognitive disorder. The reduction is in line with two other studies investigating PIMs in Swedish populations, one conducted in nursing homes in 2007 and 2013, and one conducted among the entire population aged ≥ 65 years from 2006 to 2013 [21, 22]. Both these studies were using the quality indicators published by the Swedish National Board of Health and Welfare to identify PIMs. Limited to people with major neurocognitive disorder only, a prevalence of PIM ranging from 14 to 64% was reported in a recently published review [23]. In the 12 studies identified in this review, 9 different tools were used to identify PIMs. According to results reported from a study by Renom-Guiteras et al., Sweden had a proportionally low use of PIMs compared to other European countries [24]. In that study, 49.6% people with major neurocognitive disorder in Sweden had at least one PIM; however, another tool, the European Union (7)—PIM list was used to identify PIM, which might explain the difference in prevalence with the present study.

Only one PIM group, antipsychotic drugs increased between the years, from 11.6% in 2017, to 12.3% in 2012. This PIM group was also the most extensively used in both years. However, the prevalence, in both years, is lower than results found in previous studies. For example, in another Swedish study including people with cognitive impairment living in nursing homes, antipsychotic drugs declined from 25.4% to 18.9% between the years 2007 and 2013 [25]. Major neurocognitive disorder and neuropsychiatric symptoms are associated with an increased risk of nursing home placement [26]. Consequently, the higher prevalence of antipsychotic drugs among people living in nursing homes is therefore not surprising, as these drugs are often used to control aggression or other neuropsychiatric symptoms that commonly emerge over time among people with major neurocognitive disorder [27]. In a nationwide study performed among all Danish residents aged 65 or older with major neurocognitive disorder, the prevalence of antipsychotic drug use declined between 2000 (31.3%) and 2012 (24.4%) [28]. The Danish study included both people in nursing homes and people living at home. Nevertheless, even if the prevalence of antipsychotic drugs is lower in our study than in other studies, the prevalence increased between the years. Also of concern is the association found between dementia with Lewy bodies/Parkinson’s disease dementia and PIM use. Although this can probably be explained by the higher prevalence of hallucinations and other psychotic symptoms in these subtypes compared to Alzheimer’s disease [29], people with dementia with Lewy bodies and Parkinson’s disease dementia are extremely sensitive to drugs in general, and particularly to antipsychotic drugs [30, 31]. The use of antipsychotic drugs was associated with dementia with Lewy bodies/Parkinson’s disease dementia in a study based on the same population as in the present study [32]. The use of antipsychotic drugs was also associated with a lower MMSE score in the above mentioned study [32]. This can probably explain the found association between a lower MMSE score and the use of PIMs in our study, as neuropsychiatric symptoms increase as dementia progresses [33]. The increased risk of cerebrovascular events and mortality associated with the use of antipsychotic drugs is well-documented, and guidelines recommend non-pharmacological approaches prior to considering treatment of neuropsychiatric symptoms with antipsychotics [34–36].

The second most widely used PIM group in the present study was anticholinergic drugs. This applies to both years. The decline is positive, since these drugs might produce pronounced adverse drug effects such as confusion and memory impairment among people with major neurocognitive disorder, due to cholinergic deficit [37]. In addition, anticholinergic drugs might also antagonise the potential benefits of cholinesterase inhibitors [38].

This study found that tramadol, codeine and NSAIDs decreased between 2012 and 2017. The overall prevalence of these PIMs was low in both years. The reduction is in line with guidelines and also with previous studies. For example, another Swedish study in which Hemmingsson et al. found a decrease in the use of NSAIDs and tramadol between the years 2007 and 2013 among people with and without cognitive impairment in nursing homes in Västerbotten, Sweden [39]. Results from another study investigating pain treatment in the same population as the present study indicate that the decrease of tramadol and NSAIDs in our study is probably replaced by an increase in the use of opioids and paracetamol [40].

In addition to the association between PIM use and subtypes of major neurocognitive disorders mentioned above, PIM use was also associated with younger age in this study. This association is not in line with some previously performed studies [41, 42]. One explanation could be that different criteria have been used in our study compared with previous studies, but another reason might be that prescribers have been more cautious to prescribe these drugs to older people. There have been several initiatives to improve drug treatment among old people in Sweden, such for example the development of the quality indicators from the Swedish National Board of Health and Welfare, and the introduction of medication reviews [10].

There was also an association between PIM use and using multi-dispensed drugs. This association has been observed in previous studies as well [43], and PIMs are found to be common among people using multi-dispensed drugs [44, 45]. The use of multi-dispensed drugs is believed to reduce medical errors, increase drug adherence and decrease the waste of unused drugs [46]. An association with fewer changes in drug treatment at discharge form hospital has been found [47] however, and a concern is that the physician makes fewer changes in the patients’ prescribed medicines among those using multi-dispensed drugs. Of importance is to regularly perform medication reviews in order to decrease PIMs and optimise drug treatment in this specific population.

One of the limitations with this study is that we have no data regarding diagnosis except of major neurocognitive disorder. Further, we have no data of indications for prescriptions, dosages or evaluation of treatments. Consequently, in some cases the treatment might be appropriate, such as antipsychotics in schizophrenia, hallucinations or severely aggressive behaviour. We defined drug use in this study as one or more filled prescriptions during each timeframe, but we do not know how the individuals actually used their medications. It should also be noted that the results would probably be different in this study if other criteria than the Swedish indicators had been used. A previously performed study comparing five different tools found that only 14% of PIMs were captured simultaneously. Further, the kappa coefficient varied from 0.37 to 0.75, where 0.37 referred to Swedish Indicators and Beers criteria [41]. Another limitation is that we only have information of dispensed drugs until 2017, studies with more recent data would be desirable. Furthermore, by the end of 2017 approximately 45% of all new registrations in SveDem were provided by primary care, which indicates a relatively even distribution in the reporting between primary care and specialized care. However, the reporting differs considerably between different counties in Sweden, in some counties almost all registrations are reported by specialized care units, and in some counties, there is almost none reporting at all [17]. There may be differences in cognitive function and also in drug use between people registered in SveDem and people with major neurocognitive disorder in general in the entire population. This might affect the representativeness of the population and should be borne in mind when interpreting the results. The strength of this study is that it was possible to investigate and describe PIM use in a large group of people with major neurocognitive disorder, through the use of registries.

## Conclusion

Overall, the use of PIMs decreased between the years 2012 and 2017. The increase of antipsychotic drugs and the association between PIM use and multi-dispensed drugs warrant concern.

## Supplementary Information

**Additional file 1: Table S1.** The prevalence of PIM in 2012 and 2017. Sorted by ATC code.

## Data Availability

The datasets used and/or analysed during the current study are available from the corresponding author on reasonable request.

## Abbreviations

ADR: Adverse drug reactions
MMSE: Mini Mental State Examination
PIM: Potentially inappropriate medication
SPDR: Swedish prescribed drug register
SveDem: Swedish registry for cognitive/dementia disorders
