# Supplementary material for: Prevalence of potentially inappropriate medications among old people with major neurocognitive disorder in 2012 and 2017
Source: BMC Geriatr. 2022 Jun 30;22:544. doi: 10.1186/s12877-022-03240-y (PMC9245287; doi:10.1186/s12877-022-03240-y)
Supplement: Supplementary file 1 — Additional file 1: Table S1. The prevalence of PIM in 2012 and 2017. Sorted by ATC code. [file 12877_2022_3240_MOESM1_ESM.pdf]

Prevalence of potentially inappropriate medications among old people with major neurocognitive disorder in 2012 and 2017

Additional file 1

Iris Rangfast,[1] Eva Sönnerstam,[1] Maria Gustafsson[1]

[1] Department of Integrative Medical Biology, Umeå University, 901 87 Umeå, Sweden

Corresponding Author:
Maria Gustafsson, Department of Integrative Medical Biology, Umeå University, SE-901 87 Umeå, Sweden
E-mail: maria.gustafsson@umu.se

Table S1. The prevalence of PIM in 2012 and 2017. Sorted by ATC code.

ATC code	Drug class/substances	2012 n (%)	2017 n (%)
All people		20889	35212
	**Long-acting benzodiazepines**	434 (2.1%)	511 (1.5%)
N05BA01	Diazepam	304 (1.5%)	397 (1.1%)
N05CD02	Nitrazepam	57 (0.3%)	53 (0.2%)
N05CD03	Flunitrazepam	79 (0.4%)	72 (0.2%)
	**Anticholinergic drugs**	1898 (9.1%)	2098 (6.0%)
A03AB02	Glycopyrroniumbromid	12 (0.1%)	22 (0.1%)
A03BA03	Hyoscyamine	7 (0.0%)	10 (0.0%)
A03BB01	Butylscopolamine	1 (0.0%)	1 (0.0%)
A04AD01	Scopolamine	5 (0.0%)	15 (0.0%)
C01BA03	Disopyramide	10 (0.0%)	7 (0.0%)
G04BD04	Oxybutynin	21 (0.1%)	9 (0.0%)
G04BD07	Tolterodine	169 (0.8%)	232 (0.7%)
G04BD08	Solifenacin	250 (1.2%)	241 (0.7%)
G04BD10	Darifenacin	45 (0.2%)	8 (0.0%)
G04BD11	Fesoterodine	92 (0.4%)	76 (0.2%)
M03BC01	Orphenadrine	4 (0.0%)	5 (0.0%)
M03BC51	Orphenadrine, combinations	17 (0.1%)	18 (0.1%)
N02AG01	Morphine and antispasmodics	18 (0.1%)	28 (0.1%)
N02AG02	Ketobemidone and antispasmodics	2 (0.0%)	2 (0.0%)
N04AA01	Trihexyphenidyl	6 (0.0%)	14 (0.0%)
N04AA02	Biperiden	22 (0.1%)	48 (0.1%)
N05AA02	Levomepromazine	69 (0.3%)	50 (0.1%)
N05AB04	Prochlorperazine	0	1 (0.0%)
N05AF03	Chlorprothixene	4 (0.0%)	3 (0.0%)
N05AH02	Clozapine	30 (0.1%)	57% (0.2%)
N05BB01	Hydroxyzine	554 (2.7%)	467 (1.3%)
N06AA04	Clomipramine	35 (0.2%)	75 (0.2%)
N06AA09	Amitriptyline	184 (0.9%)	314 (0.9%)
N06AA10	Nortriptyline	13 (0.1%)	12 (0.0%)
N06AA21	Maprotiline	2 (0.0%)	12 (0.0%)
R06AA02	Dimenhydrinate	6 (0.0%)	0
R06AA04	Clemastine	210 (1.0%)	170 (0.5%)
R06AD01	Alimemazine	170 (0.8%)	180 (0.5%)
R06AD02	Promethazine	68 (0.3%)	112 (0.3%)
R06AD52	Promethazine, combinations	2 (0.0%)	1(0.0%)
R06AE05	Meclozine	26 (0.1%)	46 (0.1%)
N02AX02	**Tramadol**	367 (1.8%)	114 (0.3%)

N05CM06	**Propiomazine**	608 (2.9%)	352 (1.0%)
	**Codeine**	546 (2.6%)	408 (1.2%)
N02AJ06	Paracetamol/codeine	503 (2.4%)	374 (1.1%)
N02AJ09	Codeine and other non-opioid analgesics	22 (0.1%)	14 (0.0%)
R05DA04	Codeine	22 (0.1%)	20 (0.1%)
A10BB01	**Glibenclamide**	197 (0.9%)	87 (0.2%)
	**NSAIDs**	1024 (4.9%)	941 (2.7%)
M01AB05	Diclofenac	355 (1.7%)	135 (0.4)
M01AB55	Diclofenac, combinations	26 (0.1%)	10 (0.0%)
M01AC01	Piroxicam	1 (0.0%)	0
M01AC02	Tenoxicam	3 (0.0%)	2 (0.0%)
M01AC06	Meloxicam	0	3 (0.0%)
M01AE01	Ibuprofen	253 (1.2%)	243 (0.7%)
M01AE02	Naproxen	262 (1.3%)	380 (1.1%)
M01AE03	Ketoprofen	42 (0.2%)	38 (0.1%)
M01AE14	Dexibuprofen	34 (0.2%)	21 (0.1%)
M01AE52	Naproxen and esomeprazole	2 (0.0%)	3 (0.0%)
M01AH01	Celecoxib	25 (0.1%)	23 (0.1%)
M01AH05	Etoricoxib	50 (0.2%)	99 (0.3%)
M01AX01	Nabumetone	24 (0.1%)	15 (0.0%)
	**Antipsychotic drugs**	2431 (11.6%)	4323 (12.3%)
N05AA02	Levomepromazine	60 (0.3%)	50 (0.1%)
N05AB02	Fluphenazine	1 (0.0%)	1 (0.0%)
N05AB03	Perphenazine	22 (0.1%)	5 (0.0%)
N05AB04	Prochlorperazine	0	1 (0.0%)
N05AD01	Haloperidol	407 (1.9%)	533 (1.5%)
N05AD03	Melperone	53 (0.3%)	23 (0.1%)
N05AE04	Ziprasidone	2 (0.0%)	1 (0.0%)
N05AF01	Flupenthixol	16 (0.1%)	22 (0.1%)
N05AF03	Chlorprothixene	4 (0.0%)	3 (0.0%)
N05AF05	Zuclopenthixol	70 (0.3%)	86 (0.2%)
N05AH02	Clozapine	30 (0.1%)	57 (0.2%)
N05AH03	Olanzapine	205 (1.0%)	502 (1.4%)
N05AH04	Quetiapine	318 (1.5%)	633 (1.8%)
N05AX08	Risperidone	1408 (6.7%)	2653 (7.5%)
N05AX12	Aripiprazole	44 (0.2%)	86 (0.2%)
N05AX13	Peliperidon	1 (0.0%)	1 (0.0%)

NSAIDs= Non-Steroidal Anti-Inflammatory Drugs, PIMs=Potentially inappropriate medications. The frequency and prevalence differ from the sum of PIMs within the class, because some people used more than one PIM.
